# Supplementary material for: Transcriptome Analysis of Carbohydrate Metabolism Genes and Molecular Regulation of Sucrose Transport Gene LoSUT on the Flowering Process of Developing Oriental Hybrid Lily ‘Sorbonne’ Bulb
Source: Int J Mol Sci. 2020 Apr 27;21(9):3092. doi: 10.3390/ijms21093092 (PMC7247698; doi:10.3390/ijms21093092)
Supplement: Supplementary file 1 [file ijms-21-03092-s001.pdf]

Supplementary

Table S1. Analysis of different expression patterns of carbohydrate metabolism genes

Gene ID	Name	Sor25	Sor4	LXH	cluster
First-Contig10	Sucrose synthase 2	6.00	6.84	7.88	1
Contig4657	Glucose Dehydratase	4.85	6.18	6.84	1
Contig9247	Sucrose synthase 2	5.18	5.63	6.52	1
Contig415	ADP-glucose pyrophosphorylase	5.15	5.30	6.29	1
Contig1205	Sucrose synthase 2	4.91	5.83	5.86	1
Contig909	ADP-glucose pyrophosphorylase	3.55	5.10	5.71	1
Contig12824	Sucrose synthase 2	4.09	4.65	4.97	1
Contig12275	Glyceraldehyde-3-phosphate dehydrogenase	3.04	3.49	4.11	1
Contig10659	AGPase small subunit 1	0.84	1.54	4.03	1
Contig45864	Trehalose synthase	-0.30	0.55	0.60	1
Contig87292	Sucrose synthase	-3.45	-2.29	0.40	1
Contig194	UDP-glucose 6-dehydrogenase	5.46	6.61	6.36	2
Contig9314	UDP-glucose 6-dehydrogenase	5.04	6.07	6.06	2
Contig5434	ADP-glucose pyrophosphorylase	4.47	6.53	5.17	2
Contig1420	Tonoplast monosaccharide transporter	4.95	5.85	4.96	2

Contig6978	Glycosylphosphatidylinoitol	4.96	5.18	4.89	2
Contig7715	Hexokinase	3.71	5.14	4.57	2
Contig11776	Fructose phosphotransferase	4.26	4.76	4.12	2
Contig6956	Glucose-1hDA	3.26	4.35	3.86	2
Contig13047	UDP-glucuronate 5-epimerase	2.50	4.59	3.86	2
Contig5669	Sucrose transporter	4.40	5.77	3.84	2
Contig14443	UDP-glucose 6-dehydrogenase	3.40	3.82	3.73	2
Contig8288	Glucose transporter	1.82	3.26	2.93	2
Contig13319	Sucrose transporter	1.93	3.02	2.45	2
Contig12478	Hexokinase	2.48	3.02	2.26	2
Contig46868	Hexokinase	-0.65	0.62	0.60	2
Contig50392	Glucose transporter	0.52	0.65	0.01	2
Contig21729	Glucose transporter	-1.21	-0.01	-0.88	2
Contig2653	Hexokinase	2.73	3.66	-1.07	2
Contig3235	Fructose-1,6-bisphosphata aldolase	8.73	7.82	9.20	3
Contig9581	Sucrose synthase 2	6.62	4.14	7.61	3
Contig6434	Sucrose synthase 2	6.61	5.42	7.12	3
Contig4117	Fructose-1,6-bisphosphata aldolase	6.53	4.80	5.99	3
Contig1947	Glycosylphosphatidylinoitol	5.61	4.90	5.96	3

Contig74627	Sucrose synthase 1	4.20	1.65	5.50	3
Contig16341	UDP-glucose 6-dehydrogenase	3.78	2.98	5.35	3
Contig17518	Sucrose synthase	7.61	3.48	5.27	3
Contig101	ADP-glucose pyrophosphorylase	4.93	4.36	5.06	3
Contig6569	Cell wall invertase	4.70	4.56	4.91	3
Contig1206	Sucrose synthase	4.10	2.87	4.83	3
Contig9024	Hexokinase	4.34	3.79	4.69	3
Contig5308	Glucose transporter	5.12	3.59	4.64	3
Contig19039	Glycosyltransferases	2.46	0.96	4.01	3
Contig12136	Neutral/alkaline invertase	4.05	3.79	3.88	3
Contig25130	Sucrose will eventually be exported transporters 4	6.48	3.25	3.80	3
Contig31598	Sucrose will eventually be exported transporters 2	5.36	1.67	3.76	3
Contig17969	Cell wall invertase	3.87	0.72	3.60	3
Contig15719	Glucose pyrophosphorylase	2.22	1.90	2.97	3
Contig63124	Sucrose will eventually be exported transporters 1	0.27	-1.41	2.94	3
Contig18093	Glucose transporter	1.41	1.30	2.63	3
Contig64800	Neutral/alkaline invertase	-0.51	-0.86	2.00	3
Contig7282	Vacuolar invertase	2.15	0.02	1.92	3
Contig12481	Hexokinase	1.27	0.92	1.47	3

Contig79514	Hexokinase	0.54	-1.40	0.66	3
Contig51593	Phosphoribosyltransferase	0.73	-1.91	-1.33	3
Contig85180	Hexokinase	-0.13	-2.83	-1.37	3
Contig67333	Sucrose synthase 2	0.60	-3.73	-1.63	3
Contig1844	Tonoplast monosaccharide transporter	5.58	5.34	5.13	4
Contig1555	Hexokinase	6.49	5.92	4.64	4
Contig1795	Glucose pyrophosphorylase	5.84	5.55	3.96	4
Contig10197	Terpene synthase	5.35	4.17	3.91	4
Contig14296	Sterol regulatory element binding proteins	5.28	4.05	3.48	4
Contig25586	Secreted phosphoprotein	2.50	1.62	0.45	4
Contig25871	C-glucosyltransferase	1.40	1.37	0.44	4
Contig26988	Sterol regulatory element binding proteins	2.71	2.65	0.40	4
Contig33758	Terpene synthase	0.83	0.20	-0.45	4
Contig71156	Sucrose phosphate synthase	-0.30	-1.32	-1.61	4
Contig70344	Sucrose synthase 2	0.86	-0.59	-2.74	4
Contig50329	Hexokinase	1.21	-1.76	-3.05	4
Contig49439	Terpene synthase	-0.08	-0.45	-4.80	4

Table S2. The primers sequences of candidate genes used in RT-PCR experiment

Unigene ID	Protein Description	Forward Primer Sequence (5'-3')	Reverse Primer Sequence (5'-3')	Length (bp)
Contig18745	LFY(LFY1)	CGAAGAAGGGGCTGGACTA	AGCAGTGGAAGGCGTCA	110
Contig62859	FT(FT-like protein)	GCGGCAACGATCTCAGAA	CTCAAATATGGGTTACTGGGACTC	89
Contig20174	SOC1(MADS box protein SOC1)	GGTGGTTTTCCAAACGATGA	CCTTCGTCAGTGCCTGGTGTCA	105
Contig27291	VRN(Vernalization 2-1 protein)	GCTGCCAAACCCTGGTCATCA	CATTATGCCTGGTGGTGAGTTCCC	111
Contig19513	IAA (IAA type protein)	TCACCATCGTTCTCTTGCT	CCGTTCACATACTCATTCA	114
Contig88645	ABA(CBF-like transcription factor)	CAACTCGCTGGATGGCTGCT	GCCACTCCGCCACACTCAAT	115
Contig18475	DELLA(DELLA protein)	TAGATGGCGGCGAATACCT	CACGCAGCCTCTACAGT	108
Contig17518	SS(Sucrose synthase)	CAAGAAGGTCAAGGAGCAGATG	CCGCACTAAGGAAAGCAGAG	159
Contig5669	SUT(Sucrose transporter)	TTATGGCTCTCTGCTTTGTA	TGTGCGAGTAGAAATCATTG	182
Contig13319	SUT(Sucrose transporter)	TTATGGCTCTCTGCTTTGTA	TGTGCGAGTAGAAATCATTG	182
Contig19514	HXT(Hexose transporter)	CAGCACTAAGGAAGGCAGATG	AGAAGGTCCAGGAGAAGATGAAT	157
Contig1420	TMT(transporter)	TTGGCTCTGGATCGCTATCG	CTCGCTCTCACTGTCACTCTC	94
Contig7282	VINV(Invertase)	CAGTGGTTCTTGATAGCGTTG	GAGGAGGACGATGCCATCAT	107

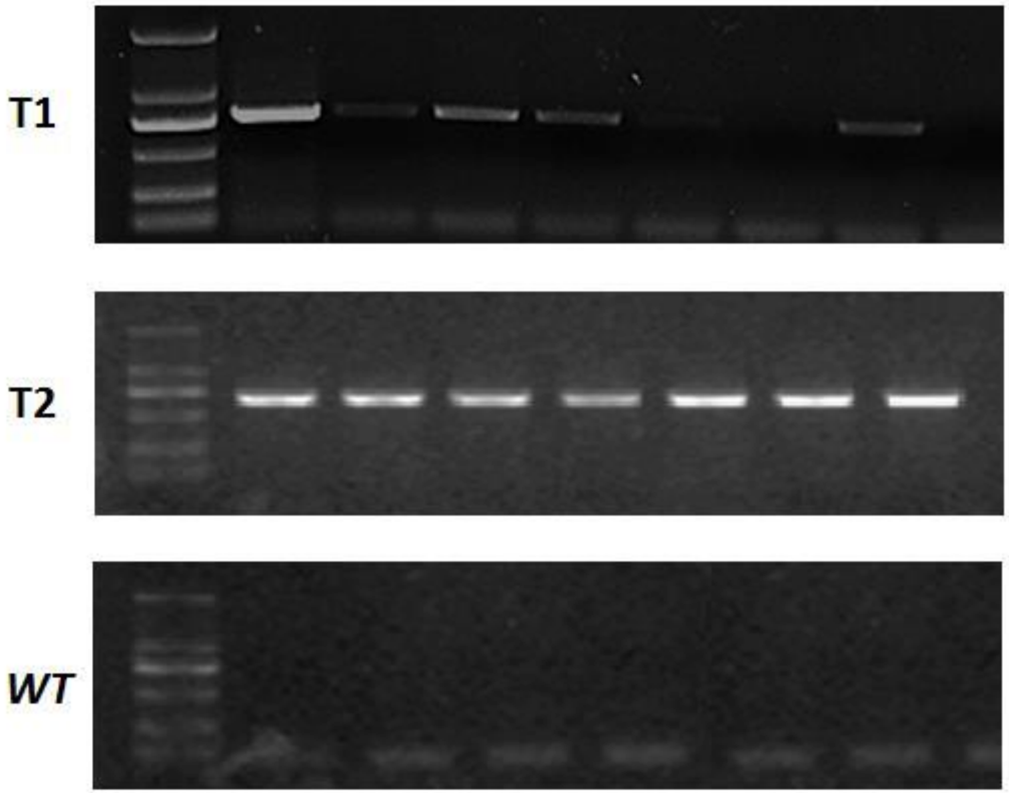

Figure S1. PCR detection of *LoSUT* gene in *Arabidopsis thaliana* transgenic plants
